# The high-intensity interval training introduced in physical education lessons decrease systole in high blood pressure adolescents

**DOI:** 10.1038/s41598-022-06017-w

**Published:** 2022-02-07

**Authors:** Marek Popowczak, Andrzej Rokita, Dawid Koźlenia, Jarosław Domaradzki

**Affiliations:** 1 Department of Biostructure, Wroclaw University of Health and Sport Sciences, Al. I.J. Paderewskiego 35, 51-612 Wrocław, Poland; 2 Department of Team Sports Games, Wroclaw University of Health and Sport Sciences, Al. I.J. Paderewskiego 35, 51-612 Wrocław, Poland

**Keywords:** Health care, Risk factors

## Abstract

Increased resting blood pressure (BP) is a risk factor for many health complications. The prevalence of elevated BP is growing among adolescents. There is a need to investigate effective ways of decreasing excessive blood pressure in this age group. The study aim was to determine the effect of 10-weeks High-Intensive Interval Training (HIIT)—Tabata protocol—introduced in physical education (PE) lessons on resting blood pressure in adolescents. The sample included 52 boys aged 16.23 ± 0.33 years body height176.74 ± 6.07 (m), body weight 65.42 ± 12.51 (kg), BMI 20.89 ± 3.53 (kg/m^2^) and 89 girls aged 16.12 ± 0.42 years, body height 164.38 ± 6.54 (m), body weight 56.71 ± 10.23 (kg), BMI 20.93 ± 3.08 (kg/m^2^) from secondary school. Based on resting BP, the fractions of boys and girls with normal BP and high BP were identified and divided into experimental (EG) and control (CG) groups. EG completed a 10-weeks HIIT program (three cycles of Tabata protocol) implemented in one PE lesson during a week. The duration of the effort was 14 min. The intensity was at 75–80% of maximal heart rate. Changes in systolic and diastolic BP after the experiment were examined. The results indicated the improvement in SBP in EG with high BP compared to the rest of the groups (average reduction of 12.77 mmHg; p < 0.0001). The EG normotensive had a statistically significant higher reduction of SBP comparing CG normotensive (average decrease of 1.81 mmHG; p = 0.0089). HIIT effectively decreases BP in adolescents. Implementing HIIT in PE lessons in secondary school is recommended to improve BP parameters.

## Introduction

Blood pressure is one of the most critical indicators of a normal cardiovascular state. Hypertension is intricately associated with numerous diseases and could lead to premature mortality^1,2^. These health problems concern not only older people but even at school age. More and more young people are at risk of blood pressure abnormalities^3,4^. Song et al.^5^ indicated a significant increase in the prevalence of high blood pressure among children and adolescents during the last 2 decades. This observation confirms the need to search for effective ways to reduce excessive blood pressure and prevent hypertension. The cardiovascular problem at a young age could become a significant health problem in the future^3,6,7^. Considering the preceding, young people should apply physical activity that leads to normotensive, which could help maintain good health status^8,9^. The need for physical activity among youth is obvious, but it is a truism to say that adolescents are generally reluctant to undertake PA in their leisure time.

Physical education (PE) lessons are relevant settings to implement physical activity for adolescents^10^. The duration of physical education lessons (usually 45 min) suggests the vital need to perform short, high-intensity efforts to increase physical activity benefits from PE lessons^11^. Bond et al.^12^ showed promising effects of High-intensity interval training (HIIT) introduced in physical education lessons. HIIT is time-efficient and effective for decreasing the risk of cardiovascular diseases (CVD)^13–15^. HIIT intervention positively affects cardiorespiratory fitness and body composition improvements in the adolescent population^16^.

However, in the HIIT effect on blood pressure, many studies concern adults^17,18^, whereas less is known about adolescents^18,19^. Morales-Palomo et al.^15^ established the positive effects of HIIT among men with metabolic syndrome. After HIIT intervention, Grace et al.^17^ showed a significant improvement in blood pressure (both systolic and diastolic) in older men leading a sedentary lifestyle. Eddolls et al.^20^ concerning HIIT effects on children and adolescents show promising data with blood pressure parameters.

Nevertheless, it is crucial to determine the possibilities and effectiveness of introducing short, high-intensity exercises during physical education lessons and their effect on blood pressure^21^. Martin-Smith et al.^22^ showed that HIIT during PE could be highly effective for cardiorespiratory status improvement among overweight adolescents. The same study demonstrated that short intensive training interventions could be as effective as prolonged ones during PE, saving time. Moreover, HIIT improves endurance, reduces body fat, increases muscle mass or cardiovascular parameters^22^. Similar effects are observed among obese adolescents, where HIIT improved anthropometric and cardiovascular parameters^22,23^. Delgado-Floody et al.^24^ found significant improvement in anthropometric and cardiovascular parameters after 28 weeks of HIIT intervention. Still, there were no significant differences in the duration of intervention between the middle and extended periods of HIIT training^25^.

The above studies showed overall positive effects on young people's health status, confirming a need to explore this area and implement HIIT in physical education lessons among adolescents^21,26^. Especially interesting to adopt the TABATA protocol^27^, a short and intensive protocol suitable for physical education lesson conditions. This effort showed a promising, broad effect on body composition and physical performance^28,29^. Less is known about the potential impacts of HIIT effects according to Tabata protocol on blood pressure among adolescents. Since hypertension is a growing health problem in the adolescent population^5^, there is a strong need to involve school youths in physical activity during PE lessons. Many evidence indicated a broadly positive effect of HIIT on health status. Therefore, this study was aimed to examine the effect of 10-weeks High-Intensive Interval Training (HIIT)—Tabata protocol^27^—introduced in physical education (PE) lessons on blood pressure in normotensive and high blood pressure adolescents concerning intersex differences.

**Figure 1:**
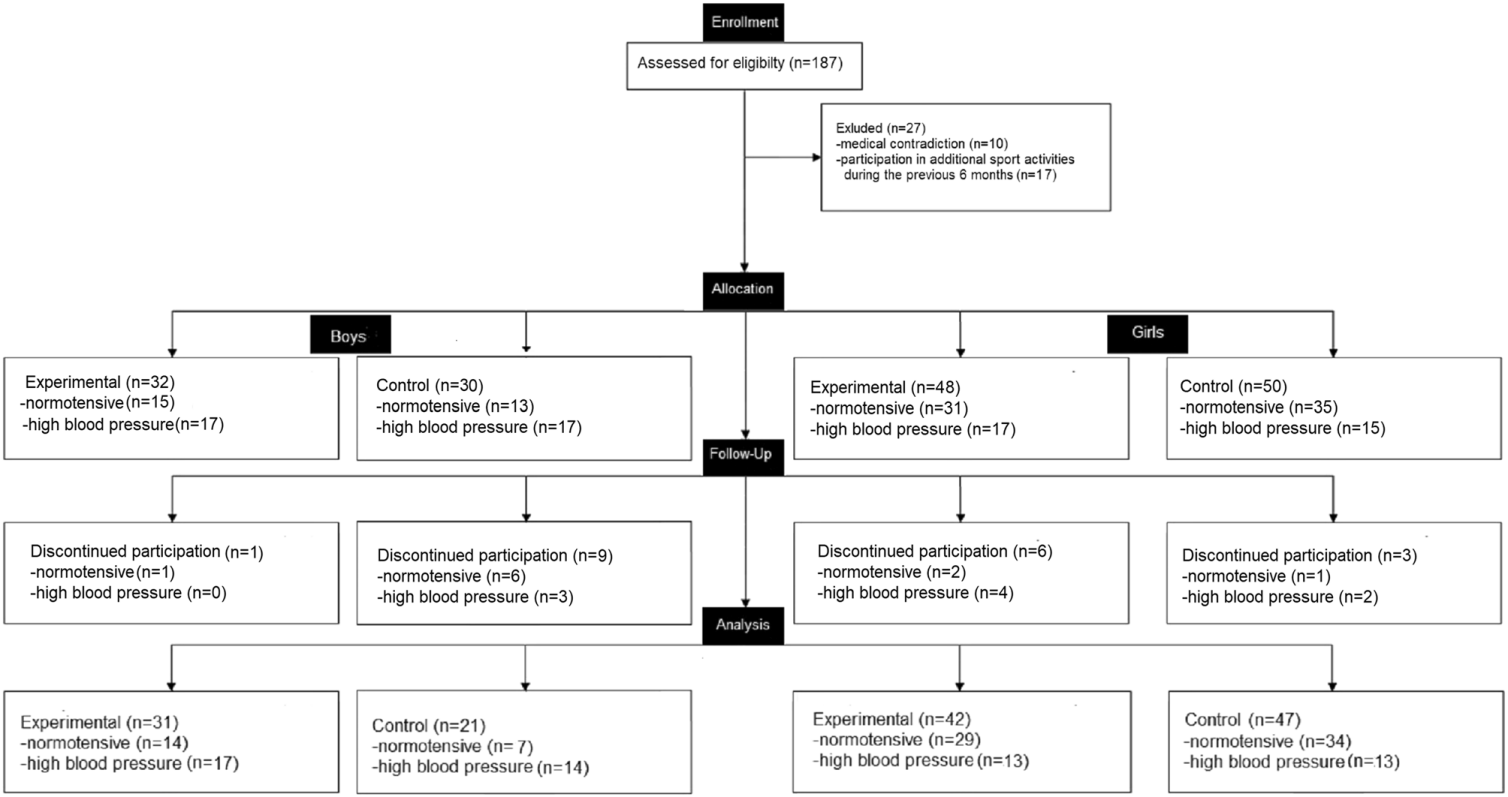
Flow-chart diagram. The flow of participants through the study.

## Methods

### Participants

Simple, non-returnable group randomization was conducted using the tool provided on the website www.randomization.com. Initially, the study sample included 187 individuals. The participants were in six separate classes, out of which three were randomly assigned to experimental and three to the control group. Each class was coded with the number from 1 to 6. Students in each class were on the same education level with the same PE education program. Before allocation to the experimental or control group, 27 subjects were excluded due to medical contradiction (n = 10) or participation in additional sports activities in the last 6 months (n = 17). The next 19 subjects were excluded during the intervention due to absence in physical education lessons. No adverse effect was noted due to HIIT intervention. The final analysis involves 141 adolescent students—52 boys aged 16.24 ± 0.33 and 89 girls 16.12 ± 0.42 from a secondary school in Wroclaw, Poland. The flow of the participants is presented in Fig. 1. The participants were volunteers, and they could quit at any time. The students and their parents or guardians were informed about the objectives and rules of the study. The school principal, parents and study participants gave written informed consent before participation.

### Measurements

Swiss anthropometer (GPM Anthropological Instruments, DKSH Ltd, Switzerland) was used for height measurements. InBody 230 machines (InBody Co. Ltd, USA), was used for bodyweight measurements. The body mass index (BMI) (kg/m^2^) was calculated based on the received data.

### Procedures

High-intensity interval training (HIIT) program based on the Tabata procedure was conducted^22^ during the one physical education lesson per week for 10 weeks. The students participated in a 14-min HIIT program. The remaining PE lessons were conducted following the PE curriculum adopted by the school for the first-year students. A detailed description of the intervention program and procedures was presented elsewhere^28^.

The tests took place in the certified Laboratory of the Physical Education Faculty at the Wroclaw University Health and Sport Sciences; ISO number: PN-EN ISO 9001:2009 and Certificate number: PW-48606-10E.

### Blood pressure measurements

All blood pressure measurements were carried out by an Omron BP710 Automatic Blood Pressure Monitor. A cuff of the appropriate size was selected based on upper arm circumference. Participants were sitting quietly for 10 min. Next, the readings were taken three times in 10-min intervals. The results introduced in the analysis were means of the three measurements.

### Identifying groups with normal blood pressure, pre-hypertension, and hypertension

The procedure of classifying the adolescents based on the systolic and diastolic blood pressure measurements met the criteria contained in the National High Blood Pressure Education Program (NHBPEP) Working Group published in the 4th Report (IVR)^29^ According to IVR, in normal blood pressure, both: systolic blood pressure (SBP) and diastolic blood pressure (DBP) are lower than the 90th percentile for both blood pressure (concerning sex and age). In a pre-hypertension state, average SBP and DBP are between 90 and 95th percentile. In hypertension state, average SBP and DBP are over 95th percentile. In addition, two stages of hypertension were distinguished: 1°—with SBP and DBP between 95th and 99th percentile and 2°—with SBP and DBP above 99th percentile.

An essential part of the procedure is using proper and adequate percentiles. Adolescents examined in this study lived in Wroclaw—a big city in the southwest of Poland. Considering that, the blood pressure standards for Polish children and youth from big cities (with over 600,000 inhabitants) were used in our study^30^. In boys, we adopted the following (mm of Hg) values: C90/SBP = 127, C90/DBP = 77, C95/SBP = 133, C95/DBP = 82. Similarly, we adopted the following (mm of Hg) values for girls: C90/SBP = 123, C90/DBP = 77, C95/SBP = 130, C95/DBP = 81. Due to the small size of groups with hypertensive adolescents, prehypertensive and hypertensive individuals were joined together. Therefore, the group was named high blood pressure. Group sizes are as follows: in experimental boys' groups, there were 14 normotensive and 17 high blood pressure subjects, and in the control group, there were 7 normotensive subjects and 14 high blood pressure subjects. In experimental girls' groups, the were 29 normotensive subjects and 13 high blood pressure subjects, and in the control group: there were 34 normotensive and 13 high blood pressure subjects.

### Intervention

In one of the three 45-min weekly PE lessons, students in the experimental groups performed a TAP^27^. In the remaining PE lessons, students improved their physical skills in various sports.

The PE lesson, in which exercises based on the Tabata protocol were introduced, was carried out according to the procedures presented by Domaradzki et al.^28^. After a 10-min standardized warm-up, students performed a 14-min TAP consisting of three sessions of 4 min each. There was a 1-min break between each session during which no exercise was performed. The Tabata protocol of each session consisted of eight cycles of two exercises. Each cycle started with a maximum intensity exercise lasting 20 s with as many repetitions as possible. This was followed by a 10-s active rest (low-intensity exercise). The following exercises were used in the first session: push-ups, high knees. In the second session—dynamic lunges, spider crawling, and in the third—plank to push-ups side squeeze. The exercises used did not require any specific exercise equipment and were aimed at working with large muscle groups. The authors prepared the exercises (for the experiment), recorded and played them back during PE lessons on the screen to accurately measure the time of exercise and rest. After TAP, flexibility and relaxation exercises were performed as a permanent program for several minutes.

The HIIT protocol was presented in video form. The intensity of exercises carried out was verified during the first PE lesson based on the procedure presented by Domaradzki et al.^28^. It was assumed that TAP exercise should be performed at high intensity, 75–80% of maximum heart rate (145–157 bpm)^22^. Experimental group EG achieved HR = 155.8 bpm (± 18.2; Cl 121; 184). In subsequent TAP sessions, exercise intensity was reached by using the video of study protocol what let to maintain the same pace of exercise, the frequency of a single movement, as obtained in the first lesson with TAP.

The control group realized a standard physical education program during the intervention.

### Statistics

Descriptive statistics were calculated: means, standard deviations (SD), and 95% coefficient intervals (CI). Changes between post-intervention and baseline measurements were calculated (Δ). Both—baseline and changes results are presented in Table 1. A three-factor analysis of covariance (ANCOVA) was used to evaluate differences between groups (sex: boys and girls, group: experimental and control, BP status: normotensive and elevated BP). Interaction terms were added and assessed too. Changes in SBP and DBP were used as dependent variables (DV). Body height and body weight were used as confounders. When significant differences were observed (significant *F*-ratio), detailed comparisons of post-hoc tests (Tukey's HSD test) were used to determine pairwise differences. The significance level was set at *α* = 0.05. All the calculations were carried out using Statistica 13.0 (StatSoft Poland 2018, Cracow, Poland).

**Table 1:** Descriptive statistics of the anthropometric and blood pressure measurements (baseline and changes (Δ)) in adolescent boys and girls from experimental and control groups by BP status.

Variable	Group	Blood pressure status	Boys		Girls	
			Baseline	Δ	Baseline	Δ
			Mean ± *SD *(95% CI)	Mean ± *SD *(95% CI)	Mean ± *SD* (95% CI)	Mean ± *SD *(95% CI)
Body height (cm)	Experiment	Normotensive	173.78 ± 5.37 170.68 to 176.88	0.26 ± 0.20 * 0.14 to 0.37	164.05 ± 6.31 161.65 to 166.45	0.14 ± 0.10 * 0.10 to 0.18
		High blood pressure	178.68 ± 6.11 175.54 to 181.82	0.17 ± 0.15 * 0.1 to 0.25	166.76 ± 5.27 163.57 to 169.95	0.17 ± 0.15 * 0.08 to 0.27
	Control	Normotensive	179.01 ± 5.54 173.89 to 184.14	0.21 ± 0.16 * 0.06 to 0.36	164.01 ± 7.41 161.43 to 166.60	0.22 ± 0.21 * 0.14 to 0.29
		High blood pressure	176.19 ± 6.17 172.63 to 179.75	0.17 ± 0.20 * 0.05 to 0.29	163.68 ± 5.87 160.14 to 167.23	0.15 ± 0.20 * 0.03 to 0.27
Body weight (kg)	Experiment	Normotensive	62.68 ± 12.37 55.54 to 69.82	− 3.69 ± 13.33 − 11.38 to 4.01	56.10 ± 8.21 52.97 to 59.22	0.40 ± 1.42 − 0.14 to 0.94
		High blood pressure	67.35 ± 14.69 59.80 to 74.90	0.42 ± 1.52 − 0.36 to 1.20	56.04 ± 5.84 52.51 to 59.56	− 0.43 ± 1.72 − 1.47 to 0.61
	Control	Normotensive	65.16 ± 7.92 57.83 to 72.48	1.13 ± 1.92 − 0.64 to 2.90	56.23 ± 9.76 52.83 to 59.64	0.23 ± 1.51 − 0.30 to 0.76
		High blood pressure	65.96 ± 12.37 58.81 to 73.10	1.21 ± 2.04 * 0.03 to 2.38	59.98 ± 17.32 49.52 to 70.45	− 0.03 ± 1.31 − 0.82 to 0.76
BMI (kg/m^2^)	Experiment	Normotensive	20.72 ± 3.76 18.55 to 22.89	− 0.28 ± 0.66 − 0.66 to 0.10	20.76 ± 2.02 20.00 to 21.53	0.11 ± 0.52 − 0.09 to 0.31
		High blood pressure	21.04 ± 4.16 18.90 to 23.18	0.03 ± 0.56 − 0.26 to 0.32	20.14 ± 1.69 19.12 to 21.16	− 0.2 ± 0.60 − 0.56 to 0.16
	Control	Normotensive	20.33 ± 2.29 18.22 to 22.44	− 0.05 ± 0.36 − 0.38 to 0.29	20.87 ± 3.18 19.76 to 21.98	0.04 ± 0.57 − 0.16 to 0.24
		High blood pressure	21.16 ± 3.30 19.26 to 23.06	− 0.01 ± 0.81 − 0.48 to 0.46	22.23 ± 5.19 19.09 to 25.37	0.13 ± 0.62 − 0.25 to 0.50
Systolic BP (mmHg)	Experiment	Normotensive	114.36 ± 7.92 109.78 to 118.93	− 1.64 ± 4.80 − 4.41 to 1.13	113.10 ± 5.07 111.17 to 115.03	− 1.9 ± 6.58 − 4.40 to 0.61
		High blood pressure	130.71 ± 11.99 124.54 to 136.87	− 13.59 ± 7.56 * − 17.47 to − 9.70	125.00 ± 9.00 119.56 to 130.44	− 11.69 ± 7.64 * − 16.31 to − 7.07
	Control	Normotensive	113.86 ± 7.84 106.61 to 121.11	2.86 ± 2.91 * 0.16 to 5.55	111.91 ± 4.79 110.24 to 113.58	2.03 ± 4.88 * 0.33 to 3.73
		High blood pressure	124.29 ± 9.05 119.06 to 129.51	− 0.71 ± 4.27 − 3.18 to 1.75	124.31 ± 8.81 118.99 to 129.63	− 2.15 ± 3.65 − 4.36 to 0.05
Diastolic BP (mmHg)	Experiment	Normotensive	70.36 ± 3.99 68.05 to 72.66	− 0.14 ± 4.13 − 2.53 to 2.24	68.45 ± 5.60 66.32 to 70.58	− 0.17 ± 9.38 − 3.74 to 3.40
		High blood pressure	77.18 ± 8.18 72.97 to 81.38	− 3.00 ± 9.30 7.78 to 1.78	78.85 ± 5.86 75.31 to 82.39	− 8.77 ± 4.95 * − 11.76 to − 5.78
	Control	Normotensive	69.43 ± 4.20 65.55 to 73.31	1.43 ± 3.46 − 1.77 to 4.63	66.94 ± 4.98 65.20 to 68.68	1.82 ± 8.38 − 1.10 to 4.75
		High blood pressure	80.07 ± 4.34 77.57 to 82.58	− 2.64 ± 5.87 − 6.03 to 0.74	78.62 ± 4.89 75.66 to 81.57	− 3.54 ± 7.28 − 7.94 to 0.86

*The statistically significant changes (Δ) p < 0.05.

### Ethical approval

The project met the ethical standards for sports medicine. The Ethics Committee of the Wroclaw University Health and Sport Sciences approved the project (ECUPE no: 19/2019). The study was conducted following the Declaration of Helsinki by the World Medical Association for research with humans.

## Results

Descriptive statistics of the parameters for boys and girls from experimental and control groups regarding BP status are presented in Table 1.

The boys and girls with high blood pressure in experimental groups were taller and heavier than normotensive peers at the baseline. Therefore, body height and body weight measurements were added to the analysis of covariance (ANCOVA) as covariates. There were no statistically significant differences between the sexes. Sex was not a factor that interacted with the effects of HIIT and blood pressure status, which confirmed the lack of statistically significant interactions (Table 2). A statistically significant intervention effect (HIIT) was only related to systolic blood pressure. *Mean square* values presented in Table 2 suggested the effect of HIIT on reducing systolic blood pressure was significant. Interaction between factors: intervention group (experimental–control) and BP status (normotensive–high blood pressure) suggested the effect of the intervention was moderated by the level of blood pressure (Table 2). Detailed comparisons in Table 3 made it possible to indicate which groups were statistically significantly different.

**Table 2:** Main effect and interaction effects of three factors: sex, intervention, and BP status on systolic blood pressure (SBP) and diastolic blood pressure (DBP) changes (Δ). The statistically significant effects in bold p < 0.05.

Effect (main and interaction)	Δ SBP			Δ DBP		
	MS	F	p	MS	F	p
Sex	0.70	0.02	0.8845	70.58	1.20	0.2756
Group (experimental vs. control)	**1691.34**	**51.60**	**0.0000**	149.07	2.53	0.1140
BP status	**1547.28**	**47.21**	**0.0000**	**775.92**	**13.17**	**0.0004**
Sex*group	27.18	0.83	0.3641	49.92	0.85	0.3589
Sex*BP_status	4.21	0.13	0.7207	87.90	1.49	0.2240
Group*BP	**347.91**	**10.61**	**0.0014**	7.26	0.12	0.7261
Sex*group*BP	13.56	0.41	0.5212	35.20	0.60	0.4408

**Table 3:** p values from Tukey's test for differences between changes (Δ) in systolic blood pressure (SBP) in experimental (E) and control (C), high (H) blood pressure, and normotensive (N) groups. The statistically significant differences in bold p < 0.05.

Variable	Groups					
	EH-EN	EH-CN	EH-CH	EN-CN	EN-CH	CN-CH
Δ Systolic BP	**p < 0.0001**	**p < 0.0001**	**p < 0.0001**	**0.0089**	0.9938	0.0988

The results presented in Table 3 indicated the statistically significant differences in changes in SBP between experimental high blood participants (EH) and the rest of the groups (average reduction of 12.77 mmHg; p < 0.0001). The experimental normotensive group had a statistically significant higher reduction of SBP comparing control normotensive groups (average decrease of 1.81 mmHG; p = 0.0089). There were no significant systolic blood pressure changes between groups.

## Discussion

A sedentary lifestyle, bad nutrition habits, and lack of physical activity are the main determinants of many health disturbances. This problem affects young people who risk many metabolic diseases by giving up physical activity. Even they could suffer abnormalities connected with high blood pressure^31^. Because of that, it is crucial to engage adolescents in physical activity, which is the best tool to defeat it. PE lessons seem to be the best settings.

However, limitations in duration lessons and the need to complete other curricular items force the introduction of effective and time-saving methods of physical activity, like HIIT, with its well-known positive effect on the cardiovascular system and endurance^13,14^. Therefore, this study aimed to determine the effect of 10-week HIIT (Tabata protocol), introduced in physical education (PE) lessons, on blood pressure in 16-years girls and boys. The study sample was at the age of dynamic adolescence, which indicated changes in body height during 10 weeks of intervention. High-intensity intermittent efforts have been demonstrated as a promising tool in improving adolescents' health^18^. This effort significantly affects metabolism even a few hours after finishing the session^32,33^. This phenomenon is associated with excess post-exercise oxygen consumption (EPOC), which demands an increased commitment of the cardiorespiratory system and has more impact on it and develops heart, blood vessels, and lunges function^34,35^.

The present study's main findings are that HIIT significantly reduced systolic blood pressure among high blood pressure adolescents and with a slightly smaller decrease in systolic blood pressure among normotensive. The obtained results show an excellent product that could be widely implemented in schools, aimed at blood pressure adjustment and CVD risk prevention^18^.

Similar to our observation, Tjonna et al.^35^ indicated a significant reduction of cardiovascular risk among overweight adolescents after HIIT program intervention through reducing blood pressure parameters. In another study, improvements in physical performance among adolescents after the HIIT program were also observed, additionally constituting a factor that keeps youth interested in physical activity^29^. Similar Farach et al.^36^ confirm positive effects of HIIT on blood pressure parameters. Moreover, it was noted improvement in fat reduction. The study by Corte de Araujo et al.^37^ showed significant improvement in systolic blood pressure comparison to continuous endurance training among obese youth.

Studies mentioned above showed excellent positive effects on adolescents. Therefore, engaging children and adolescents in any physical activity positively influence cardiometabolic risk is crucial. It is based on PE lessons, which can positively affect physical activity on health^38^. Nevertheless, intensive interval training may produce more positive effects on health than moderate-intensity training^39^. It was clearly indicated by Kouuba et al.^40^, which demonstrated more benefits from intermittent physical efforts than from continuous ones. Other studies showed similar effects between HIIT and continuous one^41^. But there must be remembered single physical education lesson is slightly short (45 min), so implementation of HIIT could be more suitable^24,28^.

Still, the duration of this type of program should typically be approximately 8 weeks or more to reach more positive effects^28,34,38^. However, some positive metabolic effects of HIIT are observed after 2 weeks^42^. Our study revealed a significant impact on SBP, with no impact on DBP among subjects with high blood pressure. A similar effect on BP through HIIT was observed by Olea et al.^43^ in an experiment conducted among adult women. Despite a different study sample, it confirms the positive effect of HIIT on blood pressure.

The effect of HIIT is more comprehensive than only BP effects. There was proof of a vast influence on plasma lipids strongly associated with cardiovascular disease risk^44^. The HIIT program duration of 8 weeks seems to be enough to achieve this effect in a group of obese young women. Furthermore, implementing HIIT in PE lessons influences the general level of physical activity. Costigan et al.^45^ indicated that adolescents were more active when they had HIIT than on other days. It is also worth noting the positive effect of HIIT regardless of body composition. It conditions the possibility of introducing this type of intervention regardless of the BMI status. It, therefore, enables a broader implementation of such efforts in various groups^22^. HIIT training seems to be an effective solution to counteract the risk of developing cardiovascular and metabolic health problems.

Even though HIIT constitutes a heavy and demanding activity, it is not only sufficient for athletes focused on developing physical performance^46^. It supports overall health effects, which could be introduced among adolescents in PE lessons at school, with success. HIIT requires a short time and enables conducting it during the 45-min lessons while being an attractive and effective way of improving health conditions.

We are aware of some limitations of our study. Due to small groups of hypertensive prehypertensive and hypertensive individuals were joined together. Only one secondary school took part in the experiment. We did not have nutrition control and average daily physical activity monitoring. There was no continuous heart rate monitoring in the following lessons.

On the other hand, this study has some strengths. It was conducted in natural school settings, indicating the possibility of implementing HIIT in the physical education program. Moreover, our study sample was heterogeneous. They were on the same level of education at a similar age.

## Conclusion

The 10-week HIIT intervention introduced in PE lessons resulted in a significant reduction of systolic blood pressure in the group with high blood pressure and slightly among normotensive groups. Changes in blood pressure values ​​were not observed in the groups following the only traditional PE program. The Tabata training positively affects blood pressure and can effectively prevent hypertension in adolescents. It suggests that implementing HIIT in physical education lessons is an effective tool for improving blood pressures parameters.

Further studies on a bigger study sample are required, and adolescents of various ages should be studied. There is a need to determine the sustainability of the induced changes. Dietary control should be considered. Participants should use accelerometers to monitor their daily physical activity in the future.

## Data Availability

The datasets generated during and/or analyzed during the current study are available from the corresponding author on reasonable request.
