# Publisher Correction: The high-intensity interval training introduced in physical education lessons decrease systole in high blood pressure adolescents

**DOI:** 10.1038/s41598-022-07364-4

**Published:** 2022-02-23

**Authors:** Marek Popowczak, Andrzej Rokita, Dawid Koźlenia, Jarosław Domaradzki

**Affiliations:** 1 Department of Biostructure, Wroclaw University of Health and Sport Sciences, Al. I.J. Paderewskiego 35, 51-612 Wrocław, Poland; 2 Department of Team Sports Games, Wroclaw University of Health and Sport Sciences, Al. I.J. Paderewskiego 35, 51-612 Wrocław, Poland

Correction to: *Scientific Reports* 10.1038/s41598-022-06017-w, published online 07 February 2022

In the original version of this Article, Jarosław Domaradzki was incorrectly listed as a corresponding author. The correct corresponding author for this Article is Dawid Koźlenia. Correspondence and request for materials should be addressed to dawid.kozlenia@awf.wroc.pl.

The original Article has been corrected.

